# Perceptions of the key components of effective, acceptable and accessible services for children and young people experiencing common mental health problems: a qualitative study

**DOI:** 10.1186/s12913-023-09300-2

**Published:** 2023-04-24

**Authors:** Susan Kirk, Claire Fraser, Nicola Evans, Rhiannon Lane, Jodie Crooks, Georgia Naughton, Steven Pryjmachuk

**Affiliations:** 1 School of Health Sciences, University of Manchester, Jean McFarlane Building, Oxford Road, Manchester, M13 9PL UK; 2 School of Healthcare Sciences, Cardiff University, Heath Park Campus, Cardiff, CF14 4XN UK; 3 The McPin Foundation, 7-14 Great Dover Street, London, SE1 4YR UK

**Keywords:** Children, Young people, Mental health, Service development, Access, Personalisation, Therapeutic relationship, Self-care, Mental health literacy, Qualitative

## Abstract

**Background:**

Children and young people’s (CYP) mental health is a major public health concern internationally and the recent Covid-19 pandemic has amplified these concerns. However, only a minority of CYP receive support from mental health services due to the attitudinal and structural barriers they and their families encounter. For over 20 years, report after report has consistently highlighted the shortcomings of mental health services for CYP in the United Kingdom and attempts to improve services have been largely unsuccessful. The findings reported in this paper are from a multi-stage study that aimed to develop a model of effective, high-quality service design for CYP experiencing common mental health problems. The aim of the stage reported here was to identify CYP’s, parents’ and service providers’ perceptions of the effectiveness, acceptability and accessibility of services.

**Methods:**

Case studies were conducted of nine different services for CYP with common mental health problems in England and Wales. Data were collected using semi-structured interviews with 41 young people, 26 parents and 41 practitioners and were analysed using the Framework approach. Patient and Public Involvement was integrated throughout the study with a group of young co-researchers participating in data collection and analysis.

**Results:**

Four key themes defined participants’ perceptions of service effectiveness, acceptability and accessibility. Firstly, open access to support with participants highlighting the importance of self-referral, support at the point of need and service availability to CYP/parents. Secondly, the development of therapeutic relationships to promote service engagement which was based on assessment of practitioner’s personal qualities, interpersonal skills and mental health expertise and underpinned by relational continuity. Thirdly, personalisation was viewed as promoting service appropriateness and effectiveness by ensuring support was tailored to the individual. Fourthly, the development of self-care skills and mental health literacy helped CYP/parents manage and improve their/their child’s mental health problems.

**Conclusions:**

This study contributes to knowledge by identifying four components that are perceived to be central to providing effective, acceptable and accessible mental health services for CYP with common mental health problems irrespective of service model or provider. These components could be used as the foundations for designing and improving services.

## Introduction

The mental health of children and young people (CYP) is a major public health concern nationally and internationally [1–6]. It has been reported that between 13 and 20% of CYP in the United Kingdom (UK), United States (US) and Canada experience mental health difficulties [7–10], with the most common diagnoses being depression, anxiety and conduct disorders [2, 8, 11]. Furthermore, socio-economically disadvantaged CYP are two to three times more likely to develop mental health problems [12]. Recent data suggests that there has been a significant increase (92%) in referrals made to children’s mental health services in England between 2017 and 2021, with a 62% increase in the number of contacts with services during the same period [13]. Indeed, research suggests that the COVID-19 pandemic has had a disproportionate impact on CYP’s mental health [14, 15].

It is recognised that mental health difficulties persist into adulthood with half of all adult mental health problems emerging before the age of 14 and threequarters before the age of 18 [16]. Mental health problems in childhood and adolescence are associated with poorer educational attainment and employment prospects; negative impacts on social relationships and increased risk of drug/alcohol use [2, 17–20]. However, there is evidence to suggest that many CYP who experience mental health problems do not seek or receive support, with reports that only around 25% of CYP who experience mental health problems actually receive support from mental health services [1, 20–24]. Studies investigating the reasons for this have identified a number of barriers: lack of information about available services; stigma associated with mental health; complex help-seeking and referral processes; high eligibility criteria; long waiting times; lack of knowledge and expertise in first contact services (i.e., education, primary care); service inflexibilities and CYP/parent’s lack of confidence in mental health services [17, 20, 25−28]. At the same time there is also a growing concern over the number of rejected referrals to services [29, 30].

For over 20 years numerous reports have described children’s mental health services in the UK as uncoordinated, fragmented, inaccessible and lacking an evidence base [1–3, 25, 31–34]. Studies examining CYP and parents’ views of services frequently identify a lack of involvement in decision making; poor continuity of support; lack of information; a clinical non-holistic approach [26, 27, 35] as well as the previously referred to barriers to accessing services. The problems identified by research and highlighted in policy documents have proved to be intractable. In response, there have been significant increases in government funding, however, this neither kept pace with demand nor has it been ringfenced to ensure it can only be used for children’s mental health services [30, 36, 37]. While multiple models of CYP mental health services have been implemented in the UK in an attempt to address service deficits (e.g., the Choice and Partnership approach (CAPA), Improving Access to Psychological Therapies Program (CYP-IAPT)), there is limited evidence of their success in ameliorating these longstanding problems [38–41]. Furthermore, little is known about the key factors that influence the effectiveness, acceptability and accessibility of services. The findings reported in this paper are from a large mixed method study that aimed to develop a model of effective and high-quality service design for CYP experiencing common mental health problems (CMHPs). For this study CMHPs were defined as anxiety, depression, obsessive-compulsive disorder (OCD), self-harm, post-traumatic stress disorder (PTSD) and emerging personality disorders. The aim of the stage of the study reported in this paper was to identify CYP’s, parents’ and service providers perceptions of the effectiveness, acceptability and accessibility of services for CYP experiencing CMHPs.

## Research methods

The stage of the study reported here used a collective case study approach to enable multiple services to be studied in their real-life context from the perspectives of different stakeholders [42]. In this study the ‘case’ was defined as a service for CYP experiencing CMHPs. Patient and Public Involvement (PPI) was central to the study with CYP and parents being involved throughout from conception to dissemination. Initially they were involved in developing the study aims and design and later CYP and parents helped in the development of participant information sheet, consent/assent forms and interview topic guides. For this stage of the study, a group of six young adults with lived experience of mental health issues were trained and employed as co-researchers to work collaboratively with the researchers in collecting and analysing data and in disseminating the research findings. We worked with the McPin Foundation (a charity that works to support young people with lived experience become involved in research) to develop a training and mentoring package for the co-researchers. The online training was facilitated by the research team and included modules on the role the of co-researcher and guidance on using lived experience; qualitative research (data collection and analysis); research integrity and ethics; and skills practice. The co-researchers conducted interviews alongside the academic researchers and were not responsible for participant recruitment or obtaining informed consent. Their role in data analysis is described below.

### Sampling and recruitment

As part of the larger study a mapping exercise had been conducted to identify out-of-hospital services provided by all sectors that were targeted at CYP aged up to 18 years with CMHPs across England and Wales. This data was collected using an online survey and internet searching. Emails with a weblink to the online survey were sent to a range of email distribution lists, organisations and individuals across the statutory and non-statutory sectors, including all NHS Child and Adolescent Mental Health Services (CAMHS) in England and Wales. The online survey was also widely publicised through social media. The mapping exercise created a sampling frame of 154 services from which 19 were purposively sampled to ensure variability in relation to their characteristics such as service provider (NHS, third sector); locality/setting (urban-rural, home-clinic, school); service user group; and mode of delivery (face-to-face, online, telephone). Of these 19 sites nine agreed to participate in the study. At each case study site the aim was to recruit a sample that reflected the particular service characteristics and which included younger and older children; parents/carers; CYP and parents/carers who had ‘dropped-out’ of services and different types of staff. From each site we aimed to recruit six to eight CYP, two to three parents/carers and two to three staff members or service commissioners who met the sample eligibility criteria (Table 1).

**Table 1:** Sample Eligibility Criteria

Inclusion Criteria	Exclusion Criteria
Current or previous service users. Children and young people (aged 8–18 years) Young adults (18–24 years). Parents/carers of children and young people using services. Front line staff delivering the service to children and young people and their families at case study sites. Service managers at the case study sites Service commissioners involved in commissioning mental health services	Any service user or parents/carers who are not able to fully understand the study and provide fully informed consent. Children aged under 8 years of age.

Potential research participants were provided with information about the study by staff from the service and adverts were also placed on some services’ websites to alert potential participants to the study. To increase the accessibility of information for CYP a video was co-designed with our co-researchers to supplement the written participant information sheets. After receiving information about the study (via participant information sheets or adverts) potential participants contacted the research team directly about taking part in the research. The researchers then discussed the study further with potential participants and arranged a convenient time for an interview. Parental permission and consent were obtained to provide CYP under 16 years old with study information and to contact them regarding study participation. Attempts were made to recruit CYP and parents who had declined support or had disengaged from the case study services by asking services to contact this group and invite them to participate in the study. However, this was unsuccessful. This issue is discussed further in the study limitations section.

### Data collection

In keeping with a case study approach multiple data collection methods were planned to be used (interviewing, observation and documentary analysis). However, the COVID-19 Pandemic led to all primary data collection (apart from one interview) being conducted remotely with ethical committee approval, and participants were given the choice between a telephone interview or a video-conferencing interview via Zoom or Microsoft Teams. Semi-structured interviews were conducted using a topic guide. The purpose of the interviews was to capture participants’ views and experiences of the service and their perceptions of its accessibility, acceptability and effectiveness. Topic guides were developed based on the study aims and in consultation with our advisory group and CYP/Parent PPI advisors (Table 2). With participant assent/consent, 96 interviews involving 108 participants were conducted (83 via Zoom/MS Teams, 12 by telephone, and one face-to-face after Covid-19 restrictions were relaxed). Of these interviews, 87 were individual interviews, eight were dyadic interviews with CYP and their parent and one was a group interview (comprising four CYP from one service). In relation to the group interview, the CYP were accustomed to group interactions using remote videoconferencing and knew one another from the service. The researcher held a preliminary discussion with the group to discuss the best way of managing turn-taking during the interview. Each CYP was given the opportunity to answer each question and they appeared engaged during the interview, listening to one another, and sharing their own experiences. Twenty-two interviews were jointly conducted by the researchers and co-researchers. Interview length ranged from 31 to 84 min (mean 56 min); with CYP interviews ranging from 31 to 65 min (mean 44 min, SD 10.12); parent interviews ranging from 32 to 73 min (mean 56 min, SD 13.73) and practitioner interviews ranging from 38 to 84 min (mean 64 min, SD 12.57). Due to COVID-19 it was not possible to conduct observations of activities at the case study sites and it proved difficult to negotiate observing any meetings held via videoconferencing. Documents about the service that were available to the research term were collected from each site to provide background information in which to contextualise the findings.

**Table 2:** Interview Topic Guides

Children and Young People	Parents	Practitioners
**Context/history** • Length of time experiencing mental health problems • Impact of mental health problems • Who helped/supported you / your family before coming to service? **Feedback on the service** • Who told them about the service • Why were they interested in going/taking part? • Did they know what to expect? Sufficient info on service provided? • Pattern / frequency of use of the service • How easy/difficult is it to access the service? • What do they like about it? • What don’t they like about it? • Do they feel it has helped them? If so, how? • Could the service be improved? If so, how? **Mental health support** • Have they used other similar services? If so, how does this one compare? • If other services are involved, who co-ordinates this – is there a key worker or care co-ordinator? • What would the ideal service look like?	**Context/history** • Length of time child / young person has experienced mental health problems • Impact of mental health problems on child and on wider family • Who helped/supported child and family before the service **Feedback on the service** • Who told them about the service • Why were they interested in going/taking part? • Did they know what to expect? Sufficient info on service provided? • Pattern / frequency of use of the service • How easy/difficult is it to access the service? • What do they like about it? • What don’t they like about it? • Do they feel it has helped their child? If so, how? • Do they feel it has helped them / wider family? If so, how? • Could the service be improved? If so, how? **Mental health support** • Have they used other similar services? If so, how does this one compare? • If other services are involved, who co-ordinates this – is there a key worker or care co-ordinator? • What would the ideal service look like?	**Role in the Service** • current role and length of time at service • specific training/education in children’s mental health **Service access and referrals** • Overview of services provided • How do the children, young people and families who access this service find out about it? • Service referral and access process • Pattern / frequency of use of the service **View of the service** • Enablers: What works well and why? • Barriers: What works less well and why? • Effectiveness: Perception of its impact on children/parents (short and long term) • Perception on the impact of the service on other support services, e.g., primary care, hospital etc. • Acceptability: Service provider views on what CYP and families like/dislike about the service • What would the ideal service for CYP experiencing common mental health problems look like?

### Data analysis

**Fig. 1:**
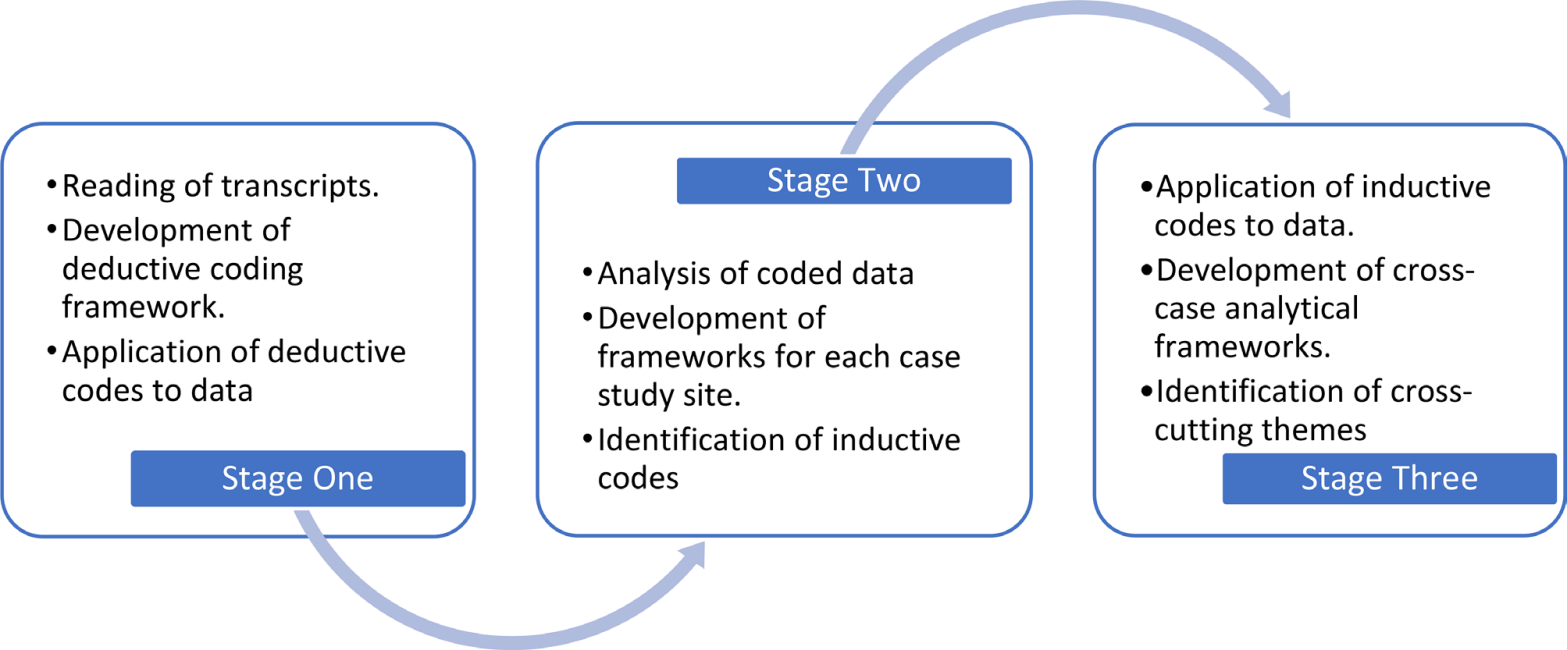
Data Analysis Process

Interview audio-recordings were transcribed verbatim and imported into NVivo 11. The Framework approach [43] guided data analysis and involves inductive and deductive coding. Initially the research team (co-researchers and academic researchers) familiarised themselves with the data by reading and discussing the transcripts. The data were then coded deductively in NVivo using a thematic framework based on the study’s aims and objectives. Examples of deductive codes include ‘access and navigating services’, ‘access barriers’, ‘access facilitators’, ‘service impact’. Following this the data were ‘charted’ by the research team to enable comparative analysis between and within case study sites. These data were then analysed inductively to identify cross-cutting themes (Fig. 1).

### Ethical issues

Ethical approval for the study was obtained from the NHS Health Research Authority (Ref: 20/SC/0174).

A range of information sheets and assent/consent forms were developed for different participant groups and for different ages of CYP. Written/verbal informed consent or assent (for children under 16 years) was obtained from participants at the time of the interview. For CYP under 16 years of age, parental consent for their child’s participation was obtained as well as the young person’s assent. However, we ensured that the final decision on participating rested with the CYP themselves. Consent/assent was regarded as a continual process with attention paid to any nonverbal signs that suggested that participants no longer wished to take part.

We were aware that there was a risk that participants might become distressed when describing difficult personal experiences. To mitigate this, we developed a distress and debrief policy for the study to ensure that participants were supported both during and after participation in the study. Protocols were also developed to manage any potential distress experienced by the researchers and co-researchers. Similarly, procedures were established for any safeguarding disclosures and the limitations to confidentiality were highlighted to participants in the information sheets.

Information and data were handled in line with the General Data Protection Regulation and Data Protection Act 2018. All services, participants and their data were anonymised. Interview transcripts were password protected and securely stored. Data extracts are anonymised using participant code numbers and are labelled CYP (child/young person), P (parent) and SP (Service Provider). The names of the case study sites have not been used in the report and instead they have been assigned a number from one to nine.

## Findings

The findings from the study are presented as a cross-case analysis, organised around the four key themes emerging from the Framework Analysis that defined the effectiveness, acceptability and accessibility of services: open access to support, therapeutic relationships, personalised support and the development of self-care skills. The characteristics of the sample and the case study sites are presented in Tables 3 and 4.

**Table 3:** Study Sample (n = 108)

Case Study	CYP	Parent/Carer	Service Provider	Total
Site 1	7	1	4	12
Site 2	5	2	9	16
Site 3	5	3	3	11
Site 4	6	0	2	8
Site 5	2	5	5	12
Site 6	6	4	5	15
Site 7	6	3	5	14
Site 8	0	7	2	9
Site 9	4	1	6	11
**Total**	**41**	**26**	**41**	**108**
**Sample Characteristics**	Female: 30 (73.2%) Male: 10 (24.4%) Gender fluid: 1 (2.4%). Age range: 9–22 years; Mean age: 17 years. Ethnicity: White British: 37 (90.2%); Asian British: 1 (2.4%); British Indian: 1 (2.4%); Black African: 1 (2.4%); White & Black Caribbean: 1 (2.4%)	Female: 23 (88.5%) Male: 3 (11.5%). Ethnicity: White British: 23 (88.5%); White other: 1 (3.8%); Black African: 1 (3.8%); Declined: 1 (3.8%)	Female: 29 (70.7%) Male: 12 (29.3%)	

### Open access to support

The openness and accessibility of mental health services were important for CYP and parents/carers when reflecting on service acceptability and effectiveness.

#### Self-referral

Self-referral enabled CYP to directly access services and bypass ‘gatekeepers’ and was valued by CYP as it avoided them having to disclose their feelings to their GP or school or reveal their difficulties to their parents. Self-referral also meant that CYP and parents/carers avoided the perceived need to present their mental health needs in a way that would trigger a referral to CAMHS (Child and Adolescent Mental Health Services) rather than be normalised or disbelieved:

*In order to get support from CAMHS, you need to, kind of, prove that you’re very sick in order to get help* (CYP04-34).

*We did take her to the GP on between probably about three or four occasions, but the GP just said, oh, well, you know, it is just sort of what happens with young people*. (P05-39)

Services recognised the value of self-referral to families in terms of improving access. As one NHS service provider described, they recognised the barriers created by expecting CYP to disclose their difficulties to ‘gatekeepers’ who may not take their concerns seriously.

*Improving access is always one which self-referral might help with. Like are young people taken seriously when they go to try and get a referral? You know, to have to have a professional referral, they have to tell the school or they have to tell a GP. …the referral process I think is definitely a barrier for some.* (SP05-10)

Indeed, self-referral was valued by services as it enhanced the quality of referral information available which could facilitate access as it enabled them to fully understand the contextual issues surrounding the CYP’s mental health problems. This reduced the possibility of CYP being assessed as not meeting the service’s eligibility criteria. As one practitioner explained:

*We like self-referrals because there’s more information and it’s bulky, there’s a bigger narrative around what might be going on. And if I’m truthful, I find the referrals that sometimes come from professionals, there’s not the information that we need.* (SP09-21)

At the same time, they were concerned about the increased demand self-referral could place on services and if this could be met. It was notable that only the third sector case study sites offered the option of self-referral. As self-referral is dependent on CYP and parents/carers being aware of the service and this route of referral, participants highlighted the importance of promoting services, particularly via schools, to ensure information was directed directly at CYP themselves, rather than relying on signposting by other services or social media which was seen as unlikely to reach most CYP.

**Table 4:** Characteristics of Case Study Sites

CASE STUDY	SECTOR	TARGET GROUP(S)	AGE RANGE	SETTING	MODE OF SERVICE DELIVERY (AT TIME OF DATA COLLECTION)
**One**	Non- statutory sector	CYP experiencing MH difficulties; LGBT & BAME CYP	10–25 years.	Community (non-health) & schools	Face-to-face & remote
**Two**	Non- statutory sector	CYP in schools/colleges assessed as having low mood	13–19 years	Schools Outreach	Face-to-face & remote
**Three**	Non- statutory sector	CYP experiencing MH difficulties	5–21 years	Community (non-health) & schools	Face-to-face & Remote
**Four**	Non- statutory sector	CYP experiencing MH difficulties	10–25 years	Online only	Remote technology
**Five**	Non- statutory sector	CYP with CMHP; specialist ADHD service.	Up to 19 years	Community health-based site & some outreach to community settings	Face-to-face & remote
**Six**	Statutory sector	Children in crisis	Up to 18 years	Outreach to home	Face to face (throughout Covid-19)
**Seven**	Statutory sector	CYP with experiencing MH difficulties; substance abuse; young offenders	Up to 18 years	Community health-based site & some outreach to community settings	Face-to-face & remote
**Eight**	Statutory sector	Carers of looked after children with developmental trauma	Up to 18 years (children)	Community (non-health)	Face-to-Face & remote
**Nine**	Non-statutory and Statutory sector partnership	CYP experiencing MH difficulties	Up to 25 years	‘One stop shop’ centre & community-based hubs (pre-C-19)	Face-to-face & remote

#### Timeliness of support

The time between seeking support and receiving support varied across the case study sites. At some sites self/professional referral rapidly led to assessment and access to therapy and support. Such accelerated access appeared to relate to assessment of a CYP’s level of risk or to be a characteristic of the service itself. Receiving support at the point of need was seen as vital as one CYP explained:

*The point of talking to someone is when you’re struggling in the moment, and I think you can get the best help when you’re struggling in the moment. And that’s what’s so good about these online services is that if I’m struggling now, I can literally just get on now and talk to someone.* (CYP04-56)

However, for most CYP attempting to access a statutory service, an assessment had led to them being placed on a waiting list, delaying the receipt of support. One CYP described a lengthy process of help-seeking and waiting lists despite their need for support being assessed as ‘urgent’:

*I saw about three different GPs before they actually referred me as urgent. … when I did get on the waiting list I had to wait for an initial assessment and then I had to wait again, months, for an actual worker to be allocated to me, even though it was urgent it said. … I think that’s really bad because I was waiting months and months on the wrong medication before I could get help* (CYP06-040).

CYP described how they felt that their mental health had worsened whilst they waited for assessment and then waited to be allocated a therapist. Waiting for support was also difficult for parents/carers to contend with and they described how communication could be poor during this period which led to feelings of distress and uncertainty over when their child would finally access support. As one parent explained this situation may only be resolved by parents/carers ‘pushing’ for appointments:

*I got really concerned about it, and so I contacted CAMHS. I tried calling, I tried emailing, but I wasn’t getting anywhere. Nobody was calling me back… there was such a gap between the initial assessment and getting that follow up, and I did chase it … we didn’t see anyone till the middle April, and that was because I pushed and pushed.* (P07-34)

Indeed, some families described being on multiple or a series of waiting lists if they were assessed as needing several services/therapies (e.g., ADHD pathways). Service providers recognised the difficulties that CYP and parents/carers experienced during this period of time and described how they had attempted to resolve waiting list issues in different ways. At one site they had started contacting CYP on their waiting list to reassure them that they had not been forgotten. Other sites had introduced a single point of access approach where referrals were assessed by a multi-disciplinary/multi-agency team which was perceived by service providers as having improved access and ensured that CYP were directed to the most appropriate service for their needs, including those who may not meet CAMHS thresholds. Indeed, some case study sites role was to support CYP on CAMHS waiting lists. However, for CYP and parents/carers the processes they encountered and navigated via a single point of access approach could feel complex and confusing if it led to multiple assessments, multiple waiting lists and transfers between different services.

#### Service ‘openness’

Once they received support, certain characteristics relating to the services ‘openness’ appeared to be important to CYP and parents/carers. They valued services that were available in the evenings and at weekends. Being able to make direct contact with practitioners when needed was also important rather than having to wait for their next scheduled appointment:

*They give you a number and you can just ring them whenever you need them and there’ll always be someone there to speak to you, (*CYP06-036)

*I literally had [practitioner] on speed dial at certain times and she’d call me back. Because I just needed that lifeline. ‘This has happened, and I really don’t know what to do’. (*P08-44)

The ‘openness’ of services and their perceived availability for support could be influenced by the convenience of location. Services that did not require travel were viewed as being more physically accessible (i.e. those delivered in homes, schools or digitally). As the study was conducted during the Covid-19 pandemic, digital provision had expanded significantly, improving access through removing travel costs/time and increasing appointment flexibility. However, accessing digital services had financial implications in relation to equipment needs (e.g., mobile phones) and internet access costs. Digital provision could also be challenging for CYP who found it difficult to express their feelings via video or in writing. Participants also described how services delivered digitally, in the home or within schools could present privacy and confidentiality issues for CYP.

Openness was also important for CYP and parents/carers in terms of being able to self-refer back into the service following discharge rather than restarting the referral process from the beginning. One parent described this as ‘a safety net’ for families:

*That reassurance again that they were there, we could always go back to them if we were really struggling. … we’re not alone, they are there if we need them … For me, it was that bit of a safety net, that comfort that, yes, we’re not struggling with this on our own, they are there* (P06-32).

### Developing therapeutic relationships

The development of therapeutic relationships was important for service engagement and appeared to be contingent on CYP and parents/carers having trust in practitioners. The development of trust appeared to be influenced by their assessment of practitioners’ specialist expertise and interpersonal qualities and the relational continuity of the service.

#### Expertise and interpersonal qualities

CYP and parents/carers described the importance of being supported by staff who they perceived as knowledgeable and skilled specialists in mental health. This instilled a sense of trust in practitioners and a sense of confidence that they would receive the support needed to improve their or their child’s mental health. However, it was important that this specialist expertise was combined with specific inter-personal qualities and skills for the development of therapeutic relationships. Practitioners who demonstrated empathy, compassion and a non-judgemental approach promoted the development of trust which helped to normalise, validate and de-stigmatise CYP’s experiences. This in turn appeared to support engagement with services and the development of positive therapeutic relationships:

*I felt like she really understood, like, the things that I was like battling with. … I think that’s why it was so positive because I didn’t feel like she was just… sometimes, with some of the people that I’ve seen for my mental health … literally just feels like I’m sat talking to this person and they’re literally sat listening to me because that’s what they’re paid to do. And I didn’t get that feeling at all with her. It felt like she wanted to be there and help me. Yeah. It didn’t just feel like a job. It felt like, you know, she actually like cared about me.* (CYP03-12).

*We really felt listened to, felt as though they’d listened to what we were saying, listened to our concerns, took it on board …recognised what we needed…. for me, as a parent, it was really reassuring. … at last, somebody’s taking the interest in getting her the help she needs. Which, for us, that was really, that was like a massive step to us … That reassurance, that point of actually we’re not alone.* (P06-32).

Participants highlighted how it was important that practitioners were perceived as approachable in order to build rapport and create a place of safety for the discussion of emotions.

*I’m the sort of person that I want like a you know, a trusting relationship with the person I’m, you know, confessing everything to, because I like I say, I don’t trust many people and I don’t talk to many people about my problems … So when I met [counsellor] I felt really comfortable, you know and not just, you know, faking trying to get better to get out of therapy with whoever it is. I actually find I’m really getting something from what she’s offering.* (CYP09-14).

CYP and parents/carers described how practitioners who they perceived as skilled in communicating with CYP in a developmentally appropriate way were able to ameliorate power imbalances and promote the development of therapeutic relationships. In addition, practitioners’ respect for CYP’s confidentiality was important in developing trusting relationships. Some CYP described incidents where they felt their confidentiality had been breached without any explanation which had led to a loss of trust in practitioners.

#### Relational continuity

Relational continuity (or practitioner consistency) was a key process through which therapeutic relationships were built and maintained. CYP valued seeing the same practitioner as this provided consistency in approach and allowed the development of trust. Some services promoted continuity by having a named worker approach or giving CYP the option of working with the same practitioner although this could create delays in receiving support if appointments with specific practitioners were not immediately available. Practitioners highlighted the emotional impact on themselves of developing therapeutic relationships and the importance for them of receiving support from their peers:

*It’s important that we feel cohesive and have that support from each other. It’s not really work you can do in isolation. …. it’s a lot for a person to contain* (SP08- 29).

### Personalisation of support

Personalisation was perceived to be essential for the provision of appropriate support and service engagement and incorporated two inter-related elements: individualisation and control.

#### Individualisation

Participants described the importance of support being tailored to their individual needs, preferences and personal context in order to improve their mental health rather than support being standardised for all service users. As one CYP explained:

*I think everybody’s experience differs … I wouldn’t want what some of my friends would want and I think that’s the main thing, like, actually that being recognised that not everybody’s going to respond well to, like, a certain kind of regime and agenda. … I don’t think that’s always recognised. … the type of therapy that I did that, like, helped me probably the most out of everything that I’ve done, that’s got me to the point where I could say that I’m much better, I was talking to my friend about it, she said I couldn’t think of anything worse than doing that…I think an ideal mental health service would be that everybody recognised that everybody is different, so not everybody’s going to respond from the same things. (*CYP03-12)

It appeared that flexibility in the delivery of support was key to ensuring that services were responsive to individual needs. Participants described the importance of services being able to focus on what was appropriate for a CYP at each session, thereby tailoring each session to their immediate concerns. As one practitioner noted, tailoring promoted engagement:

*The fact that we tailor the therapy we give to each child as well, so it’s not like they’re coming to the service and then they get, right, this is week one, we’re going to do this and that’s that. If we started the therapy sessions and we can tell that something is really bothering that child that week, then our plans kind of go out the window and we focus on the child and what they need that week, because they’re not going to be listening to you anyway if something else is going on, so it’s you need to focus on them and see what’s going on with them.* (SP05-15)

Services not being diagnostically led was felt by one practitioner to enable a more personalised and holistic approach, unrestricted by standardised protocols and pathways:

*We’re not a diagnostically led service, so we don’t do anything around diagnosis, there’s no, sort of, protocols and pathways based on that, so you’re, kind of, just interacting as a human. And, I think, seeing you as a whole and working with you in your situation I think, for some people, has been really helpful.* (SP04-31)

#### Choice and control

Being flexible and responsive was associated with giving CYP and parents’/carers’ choice and control over the support they received. Participants valued having choices over aspects such as the service setting; type of support/therapy and its duration; timing of appointments; and parental involvement as this promoted a sense of control:

*X [practitioner] kind of said, what would help, what can I do to help? And so quite quickly, we came to a point which said, how about if I ring you, you know, once a week in the morning? Well, you know, would it be better for me to you know, for you to ring me, or should we set a time, or should we? … let’s throw some ideas around. If you don’t fancy doing it, if you don’t think it’s going to work, don’t do it. But, you know, here’s what I think, you know, take it or leave it. You know, really, you know, I think I would actually struggle to think of something that could be more helpful.* (P08-41)

*We sort of just set like a plan in the first session about all the things that I wanted to talk about, and just went through them one by one … I had a lot more control over, like, when the appointments were, like when, what we talked about, what I didn’t want to talk about. And just, like, generally it was just like, I had control over it. It was up to me. Whereas with CAMHS, it’s very, like, you follow what they do. … As soon as I decided, like, oh, like I’m ready. Like I don’t think I need it anymore. She was like, completely up for it. … it was completely up to me and there was no, like, parental involvement.* (CYP03-12)

While some practitioners discussed the importance of giving CYP control and involving them in the planning and delivery of support, others described the challenges involved in balancing their assessments of a CYP’s ‘best interests’ with giving CYP choice:

*We are focused on what’s right for that young person, whether they agree or not sometimes. Most of the time they do, but whether they agree or not, we will tell them that this is in your best interests, this is why we’re doing this … I do think it’s important that we have that best interest right at the forefront of everything we do.* (SP05-07)

### Development of self-care skills

CYP, parents/carers and professionals related the acceptability and effectiveness of services with CYP and parents/carers having the opportunity to develop the underpinning knowledge and skills to manage their or their child’s mental health difficulties.

#### Development of CYP self-care skills and self-awareness

One CYP explained that knowledge development included understanding their own mental health and interactions that might trigger an emotional response:

*I just think I know more about myself than I did before. I know how to calm myself down, I know how to process things and now I can do that without even thinking about it. And I’m just a lot more open now, than I ever have been, about my past, and my mental health and stuff.* (CYP05-08)

*We learnt there was like this triangle, and it was like how your thoughts affect your feelings and your actions. So, if you stop, if you notice your thoughts or if you notice how you’re feeling then you can either try and think more positively or you can do something to make yourself feel better. And then your actions you’ve got to question whether you should be doing it or stuff like that … say me and my mum have an argument in the day then that’s going to trigger a bad day, so then I can think about what I can do to stop an argument or prevent one. So, if you notice your triggers what you can do to stop it* (CYP02-26).

Participants described being taught strategies and techniques to help emotional regulation, manage anxiety and challenge unhelpful ways of thinking. CYP, parents/carers and practitioners associated this learning with improvements in CYP mental health, self-confidence, social relationships and school engagement. CYP described how learning self-care skills improved their mental health:

*She like taught me this technique and I always use it now, like whenever I’m stressed like breathe in like calm and then breathe out, stop, and … that really helped me, and I always use that technique. I think it helps me and it helps how I feel. I feel like do you know like whenever I feel angry or I feel really stressed, … I do my breathing exercises. It just reduces it and then I feel a lot more calm.* (CYP04-44).

*They taught us techniques, which you was able to use in our own time, which helped quite drastically… One of the techniques was like, it was, I think they call it where’s the evidence? So if you’re ever feeling down, so one of the main examples they used was, if you ever have that feeling of your friends no longer want to be friends with you or something along those lines, it was, where’s the evidence to back that up? So when you come up with that in your mind, but then you can’t actually justify it, it’s able to reduce that anxiety or reduce that panic … that’s just a negative thought that you’ve come up with* (CYP02-11).

Having the opportunity to talk about their feelings was seen by some CYP as a learning activity that helped develop their communications skills and improved their mental health because they became more confident in articulating their emotions to other people rather than internalising them:

*I think especially when I was accessing the service for the first time, I really, really couldn’t talk about anything, and I think, just generally practising talking about what’s going on, it helped me to like, and I’m a much more open person now, I’m very open, and I think practising opening up and being able to put your emotions into words, that practice was really good, ‘cause I can now do that with my friends. So, it doesn’t feel like I need counselling so much as I think I needed it before, ‘cause I can talk about what’s going on now.* (CYP09-45)

*I think it’s helped me to talk instead of internalising things because I think I didn’t have them like now, I would have been keeping things inside, not telling anything to anyone. Which would probably make things worse. I guess, yeah, I guess it helps. I don’t speak to Samaritans as much anymore because I’ve got [*Site 04*].* (CYP04-056)

Participants described how it was important that sessions were young-person centred in order for CYP to engage and develop the expertise for self-care. This was associated with an approach that was informal, creative, age appropriate and importantly enjoyable.

#### Development of parent’s skills

Some services provided parents/carers with the opportunity to learn skills to support their children with anxiety, low mood and anger through peer support or more formalised teaching. One parent described how they had successfully implemented a strategy they had learned to improve communication with their child:

*We recognised that obviously she needs to manage her emotions, but also, she didn’t communicate with us effectively how she was feeling. So, we looked at communication strategies so she could really try and tell us how she’s feeling about having to tell us so much. So, we used the traffic light system to help with that. … We looked at how we progressed over the three days. Her increase in mood. Whilst she weren’t back to being the [child’s name] we knew, we noticed a change in her which was massive for us. She spent less [time in her bedroom], she’s spending more time with us downstairs.* (P06-32)

Another parent/carer described how receiving specific training on trauma-informed care had increased their understanding and skills in how to interact with their child in an appropriate way:

*I would kind of go to the first session, learn about 15 things, and immediately be thinking, oh that could help … some of that stuff really works … within a quite a short space of time he just completely was opening up, and we’re having, you know, proper conversations. And so yeah, just like it’s hard to overstate the difference it made really … I think that was what kind of got us from, you know, a 10-year-old kid that says nothing to an 11, 12-year-old that would have deep open conversations.* (P08-41).

## Discussion

This study has contributed to knowledge by identifying four dimensions that are central to participants’ perceptions of service effectiveness, acceptability and accessibility irrespective of service provider or model. These dimensions could be used as the key ‘building blocks’ for designing services for CYP with CMHP which may help address some of the long-standing problems with children’s mental health services that have proved difficult to resolve. However, this will need to be accompanied by adequate ring-fenced funding for service improvement and for training/retaining mental health staff. Moreover, in some health care systems implementing change may be constrained by health insurance and administrative issues.

The acceptability, accessibility and effectiveness of services were associated with their ‘openness’, which included the opportunity for CYP/parents to self-refer, receive support at the time of expressed need as well as service’s physical accessibility and availability. Improving access and removing the structural and attitudinal barriers that CYP and parents encounter is a government policy goal [2] and this study suggests that self-referral is one way of achieving this goal. For families self-referral enabled them to bypass the barriers created by gatekeepers, while for practitioners self-referral enhanced the quality of referral information as this came directly from CYP and parents themselves. Other studies have similarly suggested that self-referral can overcome some of the access barriers CYP experience [17, 44–47], although the role self-referral plays in improving the quality of referral information for practitioners has not been previously recognised. Self-referral is dependent on CYP/parents being aware of what services are available and the opportunity for self-referral. However, there is evidence to suggest that families lack information about service provision and the pathways to access support [17, 27, 48].

The importance that participants placed on rapid access to support in order to prevent the deterioration of CYP’s mental health resonates with previous studies [27, 49]. Indeed, it has been reported that the longer CYP spend on a waiting list the more likely they are not to engage with services [41], and that rapid access is associated with increasing their motivation to engage in therapeutic work [50]. Moreover, early intervention reduces the secondary impacts on loss of education, loss of friendships and the development of co-morbid mental health issues [51]. In our study practitioners considered that a single point of access to mental health services helped to ensure that CYP were directed to an appropriate service in a timelier way. Recent research found this approach improved the speed of the referral process but also increased the number of referrals, highlighting the need for improvements in service access to go hand-in-hand with expanding the capacity of mental health services [47]. Indeed, self-referral and other methods of improving access will inevitably increase demand, particularly given the level of unmet need, and require increased funding. However, self-referral would stop the practice of families being referred to different agencies simultaneously to see which service responds first, a practice inherently associated with increasing demand and non-attendance. Another way of meeting increased demand and reducing access barriers is through the use of unguided/guided self-care programmes [52, 53]; programmes which we suggest should be a core component of services for CYP with CMHPs. For CYP and parents in our study a single point of access approach was felt to have increased the complexity of accessing support and could lead to a series of waiting lists. Indeed, some CYP felt that they were passed between services until finally reaching the most appropriate service for their needs. This highlights the need for an integrated multi-agency approach to mental health support and for highly skilled triage decision making to ensure CYP are directed to the most appropriate service for their mental health needs [50]. Consistent with previous research, the physical accessibility and availability of services were important. Other studies have reported that flexible access and appointment times are valued by families [27, 54] and that providing services in accessible locations such as schools or via videoconferencing can improve access and engagement [17]. However, this study has highlighted that physical accessibility needs to be balanced against the potential threats to confidentiality, the financial costs and individual CYP’s preferred mode of communication. The importance of services being person-centred has been emphasised in government policy [55].

Positive therapeutic relationships were perceived to be important for service engagement and the improvement of CYP’s mental health. Such relationships were dependent on CYP/parents developing trust in practitioners which was influenced by their assessment of practitioners’ expertise and their inter-personal skills as well as the relational continuity of the service. Other studies have similarly discovered that the interpersonal qualities of practitioners are important for the development of therapeutic relationships [26, 27, 46, 56–58]. However, this study has additionally discovered that CYP and parents’ assessments of practitioners’ mental health expertise and skills are also important for developing trust. Relational continuity also plays a key role in building trust between CYP/parent and practitioner, however, there is evidence that CYP/parents often experience a lack of continuity [26, 27, 46, 57]. Our study suggests that the development of positive therapeutic relationships is an important component of an acceptable and effective service. This is supported by previous research which has reported that the quality of the therapeutic relationship between practitioner and CYP/parents promotes engagement [59, 60] and is a key predictor of positive outcomes [46]. Consequently, practitioners need to possess the personal qualities, interpersonal skills and mental health expertise to develop therapeutic relationships and the service itself has to be organised in a way to enable continuity of support.

It was important for participants that support was personalised according to individual need, preferences and context and for CYP/parents to have a sense of control over planning and delivery. Tailoring support was seen as ensuring its effectiveness and appropriateness and promoting service engagement. Others have similarly highlighted how CYP/parents value support being tailored rather than being standardised [61]. Moreover, it has been reported that such an approach can improve outcomes such as self-esteem, self-care, quality of life and mental health [48] However, organisational policies can be a barrier to such an approach, with their tendency towards inflexibility and standardisation preventing individualisation [48]. Indeed, individualisation challenges the recent implementation of the CYP-IAPT programme in England where the focus has been on creating a workforce specifically skilled in a specific modality of therapy [62]. Similarly, other studies have reported that CYP want to be involved in decision-making and have choice and control over the provision of support [26, 48, 58, 61]. Practitioner concerns regarding shared decision-making (SDM) in CYP mental health have been discovered by other researchers, with such apprehensiveness being interpreted as part of the process of integrating SDM into everyday practice and reflecting a lack of expertise in service user involvement [48, 63]. This suggests that mental health practitioners need to develop the expertise and skills in shared decision-making.

Giving CYP and parents the opportunity to develop the knowledge, skills and strategies to understand and manage their own or their child’s mental health was perceived to improve children’s mental health. This resonates with previous research which has reported that CYP/parents value learning self-care skills [54, 58, 64–66]. In addition, this study found that having the opportunity to talk about their feelings not only provided an emotional release but enabled CYP to develop their skills and confidence in communicating their feelings to others. As others have noted, programmes developing self-care skills need to use CYP friendly approaches if they are to engage CYP [54, 67].

### Strengths and limitations

This study has contributed to knowledge by illuminating how effective, appropriate and accessible mental health services can be developed for CYP with CMHPs. While the findings cannot be considered generalisable in a quantitative sense, the sample size is large (n = 108) for a qualitative study and includes CYP, parents/carers and practitioners from a range of different mental health services in England and Wales. Moreover, the study has provided insight into how CYP experience mental health services which has been relatively under-explored to date [54]. A strength of the study is the involvement of CYP and parents throughout the study from its design to the dissemination of the findings. This includes a group of co-researchers being involved in data collection and analysis. Involving young co-researchers in data generation had the benefit of providing a different perspective not only on the questions asked but also in how they were formulated due to their being more likely to share common experiences and a common language [62, 63, 68]. It was notable how the co-researchers felt more able than the academic researchers to ask probing questions of practitioners. In addition, deeper insights are developed due to the rapport developed with participants as a result of a reduced power hierarchy and shared experience [66, 68, 69]. Co-researcher involvement in data analysis helped to ensure that emerging themes were also interpreted from a lay perspective. Their interpretations of the data were informed not only by their lived experience of mental illness but also by their lived experience of being a young person. We have published a paper where we discuss our reflections on co-research and provide guidance for other researchers on how to enable and support co-researcher involvement [70].

The findings also need to be considered in relation to the study limitations. The sample of CYP and parents only includes those who have accessed services as it proved impossible to recruit ‘drop-outs’ from study sites or those who did not access or engage with mental health services. Our reliance on service providers to identify and approach CYP/parents who had ‘dropped out’ or disengaged with their own services may have contributed to this difficulty. This omission raises the possibility that the sample is skewed to those who experience fewer access barriers or are more satisfied with the services they have received. Indeed, there is evidence that a significant number of CYP ‘drop-out’ of services/treatment [71], with some groups of CYP being more likely to terminate treatment early (e.g., older age groups; those from ethnic minority groups; those from socio-economically disadvantaged households) [72–74]. Recent research has suggested that CYP ‘drop-out’ of services for a range of reasons including finding the service unhelpful, feeling that they no longer need the service or because of a lack of stability in their lives [75]. Consequently, the perspectives of CYP who ‘drop-out’ of services may have made an important contribution to the research findings.

Our sample was not ethnically representative of the population of England and Wales, with 90% of CYP and 88% of parents identifying as White British compared to 81.7% in the 2021 Census [76]. In addition, the majority of CYP and parent participants identified as female. Therefore, our findings may not reflect how CYP and parents from diverse groups perceive the effectiveness, acceptability and accessibility of services. Future research needs to explore the perceptions of these groups. Finally, conducting the study during the Covid-19 pandemic meant that we were unable to include observation as a data collection method and interviews were conducted remotely which will have influenced the data generated.

## Conclusion

This study has identified four components that from a CYP, parent and practitioner perspective are essential if a mental health service for CYP with CMHPs is to be effective, acceptable and accessible. These were open access to support, therapeutic relationships, personalised support and the development of self-care skills. Other studies have similarly highlighted the importance of these individual components, adding weight to their significance, however, in this study they have been unified and developed into a set of foundations for service design which can be tailored to a local context, and which have utility for policy and practice.

## Data Availability

The data generated and analysed during the current study are not publicly available to protect the confidentiality and anonymity of participants. Interview transcripts could identify participants and cannot be made publicly available in accordance with research ethics approval. The point of contact regarding the availability of data and materials is the corresponding author.

## List of Abbreviations

CAMHS: Child and Adolescent Mental Health Services
CMHP: Common Mental Health Problems
CYP: Children and Young People
PPI: Patient and Public Involvement
SDM: Shared Decision-Making
